# Co‐Sensitized Solar Cell Achieves 13.7% Efficiency with Bis‐Hexylthiophene Dyes

**DOI:** 10.1002/advs.202509116

**Published:** 2025-08-18

**Authors:** Heng Wu, Laia Marín Moncusí, Jing Li, Eugenia Martinez‐Ferrero, Peng Wang, Emilio Palomares

**Affiliations:** ^1^ Institute of Chemical Research of Catalonia (ICIQ)‐CERCA Avinguda Països Catalans Tarragona 43007 Spain; ^2^ Departament d'Enginyeria Electrònica Elèctrica i Automàtica Universitat Rovira i Virgili (URV) Avinguda Països Catalans 26 Tarragona 43007 Spain; ^3^ Catalan Institution for Research and Advanced Studies (ICREA) Passeig Lluïs Companys 23 Barcelona 08010 Spain; ^4^ School of Chemistry and Chemical Engineering Key Laboratory of Electrochemical Energy Storage and Energy Conversion of Hainan Province Key Laboratory of Electrochemical Energy Storage and Light Energy Conversion Materials of Haikou City Hainan Normal University Haikou 571158 China; ^5^ State Key Laboratory of Silicon and Advanced Semiconductor Materials Department of Chemistry Zhejiang University Hangzhou 310058 China

**Keywords:** charge transfer, co‐sensitization, dye sensitized solar cell, excited state, organic dye

## Abstract

Efficient anti‐aggregation and superb light harvesting in the combination of large and narrow energy gap photosensitizers play a crucial role in suppressing interfacial charge recombination in dye‐sensitized solar cells (DSCs), enabling a high open‐circuit photovoltage (*V*_oc_). Here, two organic photosensitizers, H6 and H7, featuring the bulky donor N‐(2ʹ,4ʹ‐bis(hexyloxy)‐[1,1ʹ‐biphenyl]‐4‐yl)‐2ʹ,4ʹ‐bis(hexyloxy)‐N‐methyl‐[1,1ʹ‐biphenyl]‐4‐amine and N‐(2ʹ,4ʹ‐bis(dodecyloxy)‐[1,1ʹ‐biphenyl]‐4‐yl)‐2ʹ,4ʹ‐bis(dodecyloxy)‐N‐methyl‐[1,1ʹ‐biphenyl]‐4‐amine is reported, respectively, along with bis‐hexylthiophene as the π‐linker and the electron acceptor 4‐(benzo[c][1,2,5]thiadiazol‐4‐yl)benzoic acid. Although the significantly longer alkyl chains do not change the optical energy gap, for H7, it has been able to design molecular structures that exhibit longer excited‐state lifetimes in both dye‐grafted titania and alumina films compared to its H6 counterpart. The copper‐based DSC using the longer alkyl chain‐based photosensitizer H7 achieves a high *V*_oc_ of 1.22 V, comparable to the recently explored hybrid methyl ammonium lead‐based perovskite semiconductors (PSK) in solar cells. The co‐sensitized device combined with XY1b results in an efficient DSC with an impressive fill factor of 82.1% and an excellent power conversion efficiency (PCE) of 13.7% under simulated AM1.5 G conditions at 100 mW cm^−2^. Furthermore, the best device achieves an outstanding efficiency of up to 29.7% under dim light overpassing compared to the PSK solar cells.

## Graphical abstract

The authors demonstrate that the longer excited state lifetime dye in combination with a copper electrolyte produces a high photovoltage of 1.22 V, owing to retarded interfacial charge recombination. The co‐sensitized solar cell achieves efficiencies of 13.7% under full sunlight and 29.7% under LED light.

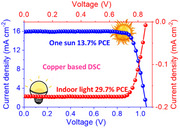

## 1 Introduction

The development of renewable and sustainable photovoltaic (PV) technology is a pressing issue for reducing global primary energy consumption in the current context of global warming and increasing energy demand.^[^ ^1^ ^]^ Unlike other photovoltaic approaches, such as perovskite solar cells,^[^ ^2^, ^3^, ^4^ ^]^ organic solar cells,^[^ ^5^, ^6^, ^7^ ^]^ and silicon solar cells,^[^ ^8^, ^9^, ^10^ ^]^ dye‐sensitized solar cells^[^ ^11^, ^12^, ^13^, ^14^, ^15^, ^16^, ^17^, ^18^, ^19^, ^20^, ^21^ ^]^ (DSCs) exhibit lower power conversion efficiencies (PCE) under standard AM 1.5 G conditions. However, with their tunable color palettes and diaphaneity,^[^ ^22^, ^23^, ^24^, ^25^ ^]^ DSCs are well‐suited for commercial applications in ambient light conditions. Recently, a high PCE of 37%^[^ ^26^ ^]^ under ambient light has been reported, indicating the potential of this technology as a power source for charging portable and low‐power consumer electronics, which may represent more than 70 billion dollars in 2032. Yet, further development must address drawbacks such as instability caused by using volatile iodine‐iodide electrolytes to regenerate the photoactive dye.^[^ ^27^ ^]^ Researchers have found that earth‐abundant copper complexes with high redox potentials can enable fast dye regeneration with relatively low driving force, significantly yielding a high photovoltage (*V*_oc_) exceeding 1.0 V.^[^ ^28^, ^29^, ^30^, ^31^, ^32^, ^33^, ^34^, ^35^ ^]^ Imahori outlined the key requirements and future prospects of copper‐based redox shuttles for achieving PCE exceeding 15%, as revealed through mechanistic insights and the photovoltaic performance of copper‐based DSCs.^[^ ^36^ ^]^ Ren and co‐workers reported a DSC based on the large energy‐gap dye MS5, paired with a copper electrolyte, achieving a record *V*_oc_ of 1.24 V. The MS5‐based DSC, co‐sensitized with the narrow‐energy‐gap dye XY1b, demonstrates an impressive PCE of 13.5% under standard AM 1.5 G sunlight conditions and remains stable for 1000 h under light soaking at ambient conditions.^[^ ^37^ ^]^

Additionally, DSCs utilizing copper redox mediators have been improved by Ren, reaching a benchmark PCE of 15.2% with a pre‐absorption strategy. This strategy effectively restrains the unfavorable aggregation of dye molecules, resulting in an ordered self‐assembled monolayer.^[^ ^38^ ^]^ Conversely, to ensure high performance in metal‐free organic photosensitiser DSCs prepared with copper electrolytes, the photosensitising dye must possess high molar extinction coefficients and panchromatic absorption to generate charge carriers and form a compact self‐organised molecular layer on titania that suppresses interfacial charge recombination. Furthermore, the dye should have a highest occupied molecular orbital (HOMO) energy level that aligns with the redox potential of the copper complex to provide sufficient driving force. It is also essential to modify the flexible side chains on the photoactive backbone of the sensitizer to mitigate the unfavorable intermolecular π–π stacking of dye molecules grafted onto the surface of titania, which is typically thought to lead to dissipative exciton annihilation and, thus, poor device performance.

**Figure 1:**
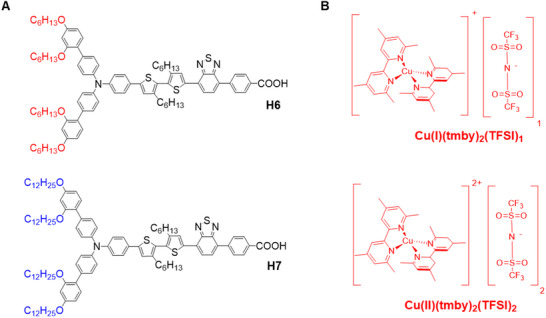
A) Chemical structures of dyes H6 and H7 with different end alkyl chains. B) Molecular structures of copper complex ([Cu(II/I)(tmby)_2_](TFSI)_2/1_ (tmby = 4,4ʹ,6,6ʹ‐tetramethyl‐2,2ʹ‐bipyridine, TFSI = bis(trifluoromethylsulfonyl)imide)).

Herein, we report two new organic photosensitizers **H6** and **H7** (**Figure** **1**A), formed by the bulky auxiliary donor *N*‐(2ʹ,4ʹ‐bis(hexyloxy)‐[1,1ʹ‐biphenyl]‐4‐yl)‐2ʹ,4ʹ‐bis(hexyloxy)‐*N*‐methyl‐[1,1ʹ‐biphenyl]‐4‐amine (BP_C6_‐DPA), and *N*‐(2ʹ,4ʹ‐bis(dodecyloxy)‐[1,1ʹ‐biphenyl]‐4‐yl)‐2ʹ,4ʹ‐bis(dodecyloxy)‐*N*‐methyl‐[1,1ʹ‐biphenyl]‐4‐amine (BP_C12_‐DPA), respectively, combined with the bis‐hexylthiophene as π linker, and the electron acceptor 4‐(benzo[*c*][1,2,5]thiadiazol‐4‐yl)benzoic acid (BTBA). The different alky chains presented here aim to disclose the effect of ancillary electron donors on self‐assembly kinetics and charge recombination. **H7** sensitizer can be employed for the fabrication of efficient DSCs with Cu(II/I) redox mediator (Figure 1b) of [Cu(II/I)(tmby)_2_](TFSI)_2/1_ (tmby = 4,4ʹ,6,6ʹ‐tetramethyl‐2,2ʹ‐bipyridine, TFSI = bis(trifluoromethylsulfonyl)imide), achieving a high *V*_oc_ of 1.22 V. Furthermore, we performed femtosecond fluorescence decay, nanosecond transient absorption spectroscopy, charge extraction, and transient photovoltage decay measurements to reveal the impacts of the variation of alkyl chains on the charge separation yield, and the interfacial charge recombination rates. Femtosecond fluorescence decay manifests that **H7,** featuring much bulky and longer alkyl chains of structure, suppresses the immoderate aggregation‐induced excited‐state quenching, elongating the excited‐state lifetime, which results in the expected increase in solar cell efficiency.

## 2 Results and Discussion

### 2.1 Synthesis and Opto‐Electronic Properties of the Photosensitizers

The synthetic routes followed to prepare the wide‐energy‐gap dyes **H6** and **H7** are described in Scheme S1 (Supporting Information). Briefly, compound 3 bis‐hexylthiophene was converted to compound 4 via the palladium‐catalyzed direct arylation reaction. Subsequently, Suzuki‐Miyaura cross‐coupled the monobromide compound 5 and the bulky triphenylamine boronic acid pinacol ester produced the butyl esters compounds 6 and 7, respectively. Finally, the carboxylic esters were treated with strong base potassium hydroxide, and the hydrolysates were acidified with a diluted hydrochloric acid aqueous solution to give the desired dyes **H6** and **H7**. For details on synthesis and structural characterization, see Scheme S1 (Supporting Information) and Appendix.

**Figure 2:**
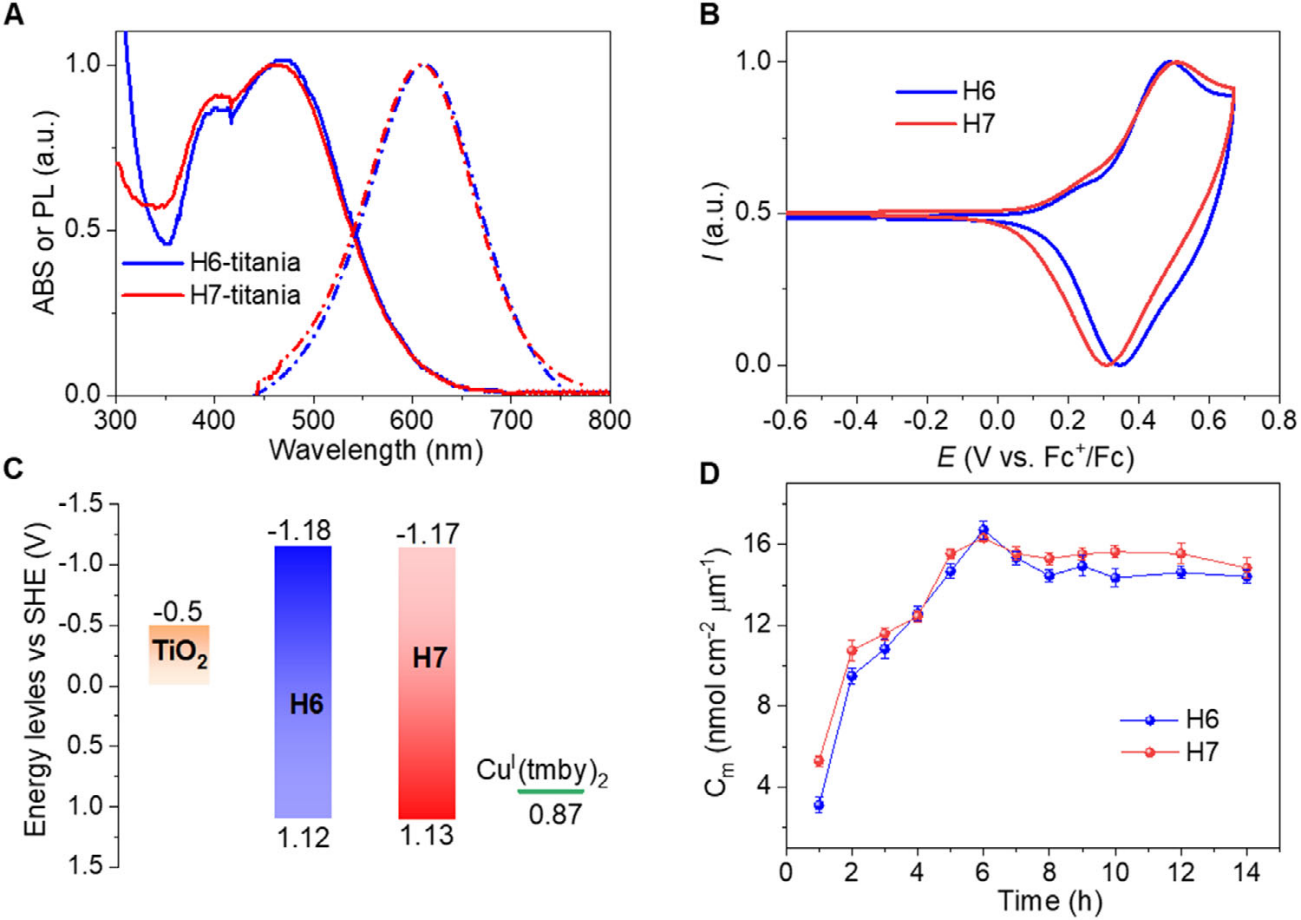
Opto‐electronic Properties of the Photosensitizers. A) UV–vis absorption and photoluminescence spectra of **H6** and **H7** grafted at 4 µm‐thick transparent TiO_2_ films. Excitation wavelength: 405 nm. B) Cyclic voltammogram curves of dyes adsorbed on 4 µm thick mesoscopic TiO_2_ films supported by FTO electrodes in acetonitrile using a 0.1 m tetrabutylammonium hexafluorophosphate (TBAPF_6_) solution as supporting electrolyte. The scan rate is 50 mV s^−1^, and Fc/Fc^+^ is the external reference. C) Energy levels diagram of TiO_2_, dyes, and [Cu(I)tmby)_2_][TFSI]. D) Stacked bar chart of dye load amount (*C*_m_) as a function of staining time for dye‐grafted 4‐µm‐thick transparent TiO_2_ films.

In contrast to MS5, **H6** and **H7** exhibit the expected intense absorption peak ≈400 nm with a high molar extinction coefficient of 3.13 × 10^4^ mol^−1^ cm^−1^ L at 445 nm, and 3.04 × 10^4^ mol^−1^ cm^−1^ L at 444 nm, respectively, increasing the blue light harvesting (Figure S2, Supporting Information). The UV–Visible absorption spectra of both dyes adsorbed on 4 µm thick transparent titania (TiO_2_) films are presented in **Figure** **2**A and listed in **Table** **1**. The maximum absorption wavelength is 472 and 466 nm for dye **H6** and dye **H7**, respectively. The steady‐state photoluminescence (PL) spectra for **H6** and **H7** coated titania films (Figure 2A, dot lines) exhibit an emission spectrum similar for both dyes that mirror the absorption spectrum, showing significant Stokes shifts, owing to the vibrational and torsional transitions from excited states to ground state. The fluorescence lifetime of the dyed titania substrates is 158 ps for **H6**, shorter than the 182 ps estimated for **H7** (Figure S3, Supporting Information), probably due to the longer alkyl chain that retards non‐radiative recombination.

**Table 1:** Electronic absorption and electrochemical properties of dyes **H6** and **H7**.

Dyes	*ε*[× 10^4^ M^−1^ cm^−1^] ($\lambda_{\mathrm{abs},\max}^{\mathrm{sol}}$) ^a)^	$\lambda_{\mathrm{abs},\max}^{\mathrm{film}}$ ^b)^[nm]	*E*_0−0_ ^c)^[eV]	*E*_ox_ ^d)^[V vs. Fc^+^/Fc]	*E*_ox_ ^e)^[V vs. SHE]	*E*_red_ ^f)^[V vs. SHE]
**H6**	3.13 (445 nm)	472	2.29	0.491	1.11	−1.18
**H7**	3.30 (444 nm)	466	2.29	0.510	1.12	−1.17

^a)^ Measured from 0.1 m dyes solution in THF, presented in Figure S2 (Supporting Information);

^b)^ Measured from dyes anchored on the 4.0 µm‐thick transparent TiO_2_ films;

^c)^ Calculated from the absorption onset wavelength (*λ*_onset_) of the corresponding dye using the equation: *E*_0−0_ = 1240 / *λ*_onset_. *λ*_onset_ determined by the intersection of normalized absorbance and emission spectra;

^d)^ Measured from the cyclic voltammogram of the corresponding dye adsorbed on a 4.0 µm‐thick transparent TiO_2_ film in a 0.1M tetrabutylammonium hexafluorophosphate (TBAPF6)/acetonitrile supporting electrolyte vs. ferrocene as internal standard;

^e)^ The potential vs. ferrocene was converted to that vs. standard hydrogen electrode (SHE) by adding 0.624;

^f^ ^)^ Calculated from equation: *E*_red_ = *E*_ox_− *E*_0−0_.

Figure 2B displays the cyclic voltammetry measurements of the two dyes anchored on the titania films in a three‐electrode system to disclose the energy level alignments. The first oxidation potentials (*E*_ox_) of **H6** and **H7** on titania film are 1.11 and 1.12 V vs. the standard hydrogen electrode (SHE), respectively, being more positive than the redox potential of [Cu(I)(tmby)_2_][TFSI] (0.87 V vs. SHE), which enables a sufficient driving force for hole injection. The zero‐zero exciton energies (*E*_0−0_) determined from the intersection of normalized absorbance and emission dyes on titania films (Figure 2A) are 2.30 eV for both **H6** and **H7**. The reduction potentials (*E*_red_) of the two dyes are more negative than the conduction band edge of TiO_2_ (–0.5 V vs SHE), being –1.18 V and –1.17 V vs SHE for **H6** and **H7**, respectively, which engenders sufficient electron injection driving force (Figure 2C).

### 2.2 Self‐assembly Kinetics on Dye Grafted TiO2 Film

To investigate the effects of alkyl chain length and dye aggregation on interfacial kinetics, we measured the time dependence of the number of moles of adsorbed dye molecules per geometric area and unit film thickness (*C*_m_) during the staining process. As shown in Figure 2D, the absorption process increases rapidly during the first 4 h of staining for both **H6** and **H7**, reaching a high value of *C*_m_. Beyond this point, absorption fluctuates over time due to changes between the adsorption and desorption dynamic equilibrium of dye molecules on the TiO_2_ surface. Therefore, controlling the staining time is essential for achieving a denser molecular packing pattern. The peak value of *C*_m_ is 16.83 nmol cm^−2^ µm^−1^ for dye **H6** and16.19 nmol cm^−2^ µm^−1^ for dye **H7**, both occurring at 6 h. Although the *C*_m_ of both dyes is comparable, the longer alkyl chains and larger molecular structure of **H7** result in more compact packing patterns, thus enhancing the surface.

### 2.3 Photoinduced Charge Transfer at the TiO2/Dye Interface

**Figure 3:**
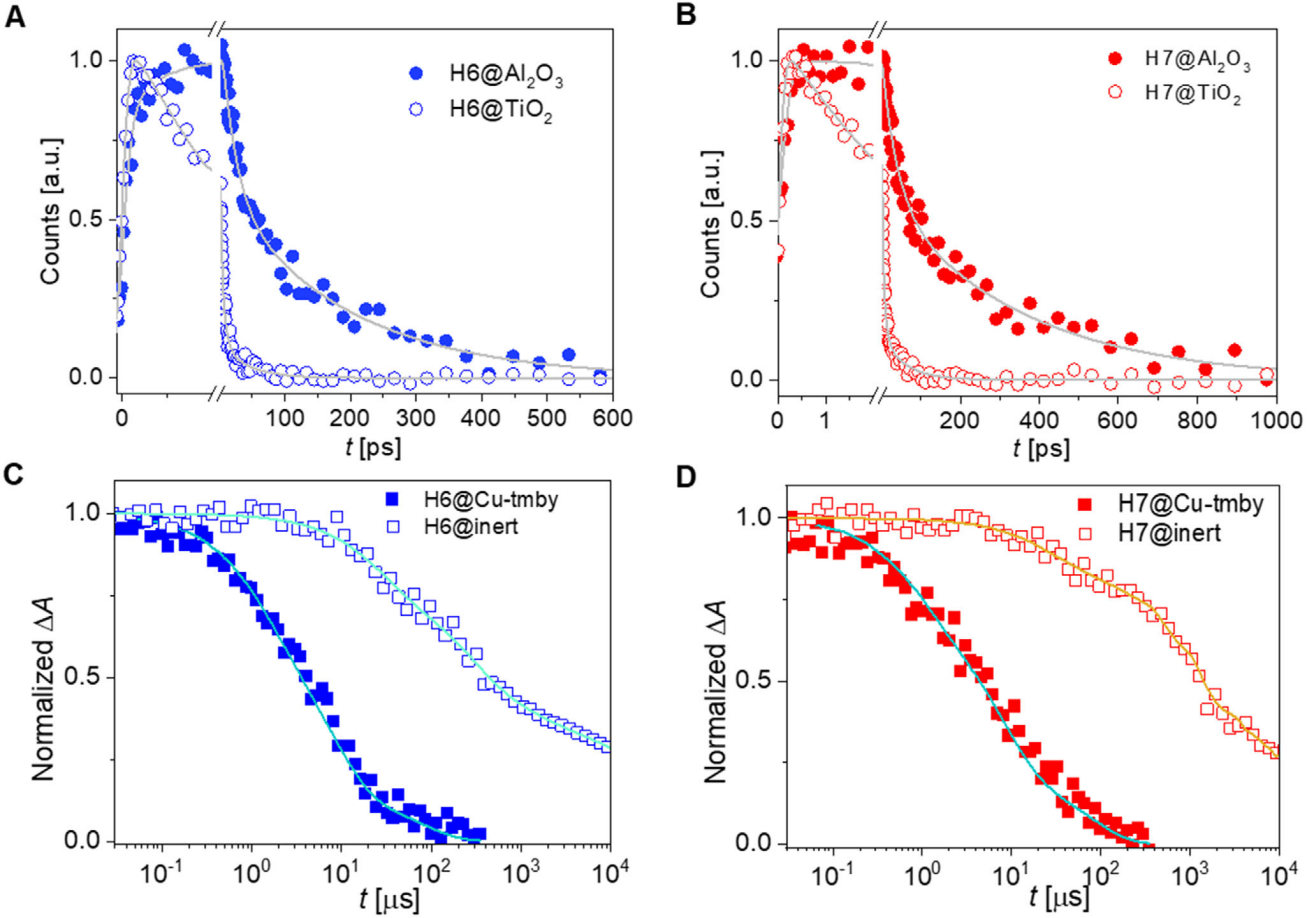
A,B) Up‐converted PL traces of **H6** and **H7** grafted semi‐transparent Al_2_O_3_ and TiO_2_ films in combination with copper electrolyte. The gray lines are multiexponential fittings, and the fitting parameters are tabulated in supporting information (Table S1, Supporting Information). Excitation wavelength: 490 nm. C,D) Normalized absorption transients of excited‐state **H6** and **H7** grafted on titania in junction with an inert acetonitrile electrolyte composing of 0.6 m *N*‐methylbenzimidazole (NMB) and 0.1 m Lithium bis(trifluoromethanesulfonyl)imide (LiTFSI) or copper electrolyte. Excitation wavelength: 525 nm for **H6** and **H7**. Pulse fluence: 35 µJ cm^−2^. Probe wavelength: 1300 nm.

To further investigate the interfacial charge transfer dynamics, we have estimated the yield of electron injection (*ϕ*_ei_) from the nonequilibrium hot, excited state of the dye molecule to titania nanocrystals by measuring the time‐resolved fluorescence decay^[^ ^39^, ^40^, ^41^, ^42^, ^43^ ^]^ on dyed titania and alumina films (**Figure** **3**A,B). It should be noted that the dyed nanocrystal oxide films are in conjunction with a Cu‐tmby electrolyte for dummy transparent DSC fabrication. Time constants and amplitudes employed to fit the fluorescence decays are listed in Table S1 (Supporting Information). The excited state lifetime ($\bar{\tau }\left(A\right)$) of dye‐anchored alumina dummy cells is 126 ps for **H6**, and 205 ps for **H7**, much longer than that calculated on dyed titania counterparts ($\bar{\tau }\left(T\right)$): 6.8 ps for **H6** and 10.5 ps for **H7**. The shortened fluorescence lifetime of dyed titania samples is inherently associated with efficient electron injection from excited‐state dye molecules to the conduction band of titania. The fluorescence ϕ_ei_ values of the equilibrium excited states, estimated with Equation (4), are 96% for **H6** and 95% for **H7**.

Furthermore, transient absorption spectroscopy was performed to monitor the kinetic decays probed at 1300 nm (Figure 3C,D), aiming to assess the nanosecond to millisecond kinetics of interfacial charge‐transfer reactions involving photogenerated holes on oxidized dye molecules with electrons in titania, or with both electrons in titania and copper(I) ions in the copper electrolyte.^[^ ^44^, ^45^ ^]^ The detailed protocols are included in the Experimental Section (Photophysical Measurements and Data Analysis). For the dye‐grafted transparent titania films in contact with an inert electrolyte composed of 0.6 m *N*‐methylbenzimidazole (NMB) and 0.1 m lithium bis(trifluoromethanesulfonyl)imide (LiTFSI), slow kinetic traces intrinsically correlated with the interfacial charge recombination of photo‐oxidized dye molecules with electrons in titania occur in the millisecond time region, with half‐reaction time constants $\left(t_{1/2}^{\mathrm{inert}}\right)$ being 423 µs for **H6** and 1280 µs for **H7**. The higher $t_{1/2}^{\mathrm{inert}}$ value of **H7** is likely due to a more significant distance from the positive charge on photo‐oxidized dye to the titania surface or due to the smaller tile angle of **H7** molecules adsorbed on the surface titania^.[^ ^46^, ^47^ ^]^ Owing to hole injection from photo‐oxidized dye molecules to copper(I) ions, accelerated kinetic decays of dyed titania films with copper electrolyte take place in the microsecond time domain, with time constants $\left(t_{1/2}^{\mathrm{Cu}}\right)$ being 4.5 µs for the **H6** sample and 5.3 µs for the **H7** counterpart. Overall, the hole injection yields (ϕ_hi_) determined via Equation (5) are close to unity for both dyes.

### 2.4 Photovoltaic Properties of High *V*_oc_ DSCs

**Figure 4:**
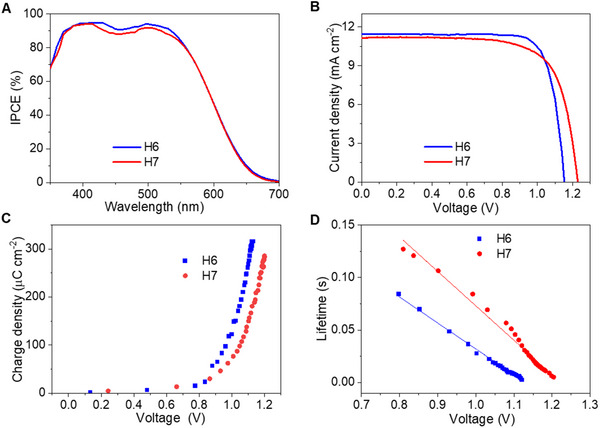
A) Plots of incident photon‐to‐electron conversion efficiency (IPCE) as a function of wavelength. B) Current density−voltage (*J*−*V*) curves at an irradiance of 100 mW cm^−2^, AM1.5G conditions. C) Plots of charges density extracted from dye‐grafted titania films as a function of voltage. D) Plots of half‐lifetimes of electrons stored in titania as a function of voltage. The recombination rate is the reciprocal of lifetime. Fitting the data, the recombination rate of **H6** is 4.1 s^−1^, much twice than that of **H7** (1.9 s^−1^).

We employed the dense bilayer dye‐grafted titania film to fabricate DSCs with a copper electrolyte. The details for device manufacture are described in the Experimental Section. The photocurrent action spectra of DSCs are shown in **Figure** **4**A. The incident photon‐to‐electron conversion efficiency (IPCE) peak values are over 90% for **H6** and **H7**, which agree with the comparable yields of electron injection and hole injection estimated with time‐resolved photophysical measurements. The spectra for both dyes show the same shape, covering from 400 to 700 nm. The onset wavelength of photocurrent response is ≈700 nm for both two dyes, which is in good accordance with the absorption spectra of both dye‐grafted titania films. The photocurrent density−voltage (*J*−*V*) curves of **H6** and **H7** cells were measured under the illumination of 100 mW cm^−2^, simulated AM1.5G sunlight (Figure 4B). The **H6** cell affords a short‐circuit photocurrent density (*J*_sc_) of 11.41 mA cm^−2^, an open‐circuit photovoltage (*V*_oc_) of 1151 mV, and a fill factor (FF) of 76.2%, yielding a good PCE of 10.0%. Significantly, the **H7** cell has an improved *V*_oc_ of 1220 mV, the *J*_sc_ of 11.24 mA cm^−2^, and the FF of 72.5%, producing a PCE of 9.8%. The integrated current density derived from the IPCE (Figure 4A) is close to the *J*_sc_ determined from *J‐V* curves.

By recording *J*−*V* curves of cells with **H6** and **H7** under a series of irradiances attenuated with neutral metal meshes, we could plot *V*_oc_ as a function of *J*_sc_. Obviously, H7 displays an enlarged *V*_oc_ at a certain *J*_sc_ than that of **H6** (Figure S4, Supporting Information). We then implemented the charge extraction (CE) and transient photovoltage decay (TPD)^[^ ^48^, ^49^ ^]^ measurements to obtain more information about the underlying interfacial energetics and kinetics behind the variation of *V*_oc_. The photovoltage versus charge plot (Figure 4C) shows that the higher *V*_oc_ of the devices employing **H7** compared with **H6** is in good agreement with these charge extraction measurements. The lower *V*_oc_ of the devices based on **H6** is directly related to the electron lifetime, in agreement with previous measurements. From Figure 4D it becomes clear that the recombination rate of **H6** cells is almost 2 times faster than that of **H7**. The higher recombination rate is thus responsible for the 70 mV difference in *V*_oc_ for these dyes. We suggest that the presence of much longer alkyl chains close to the TiO_2_ surface could play a role in accelerating the recombination rate in concomitance with its lower driving force for regeneration.

### 2.5 Highly Efficient Co‐Sensitized Solar Cells

**Figure 5:**
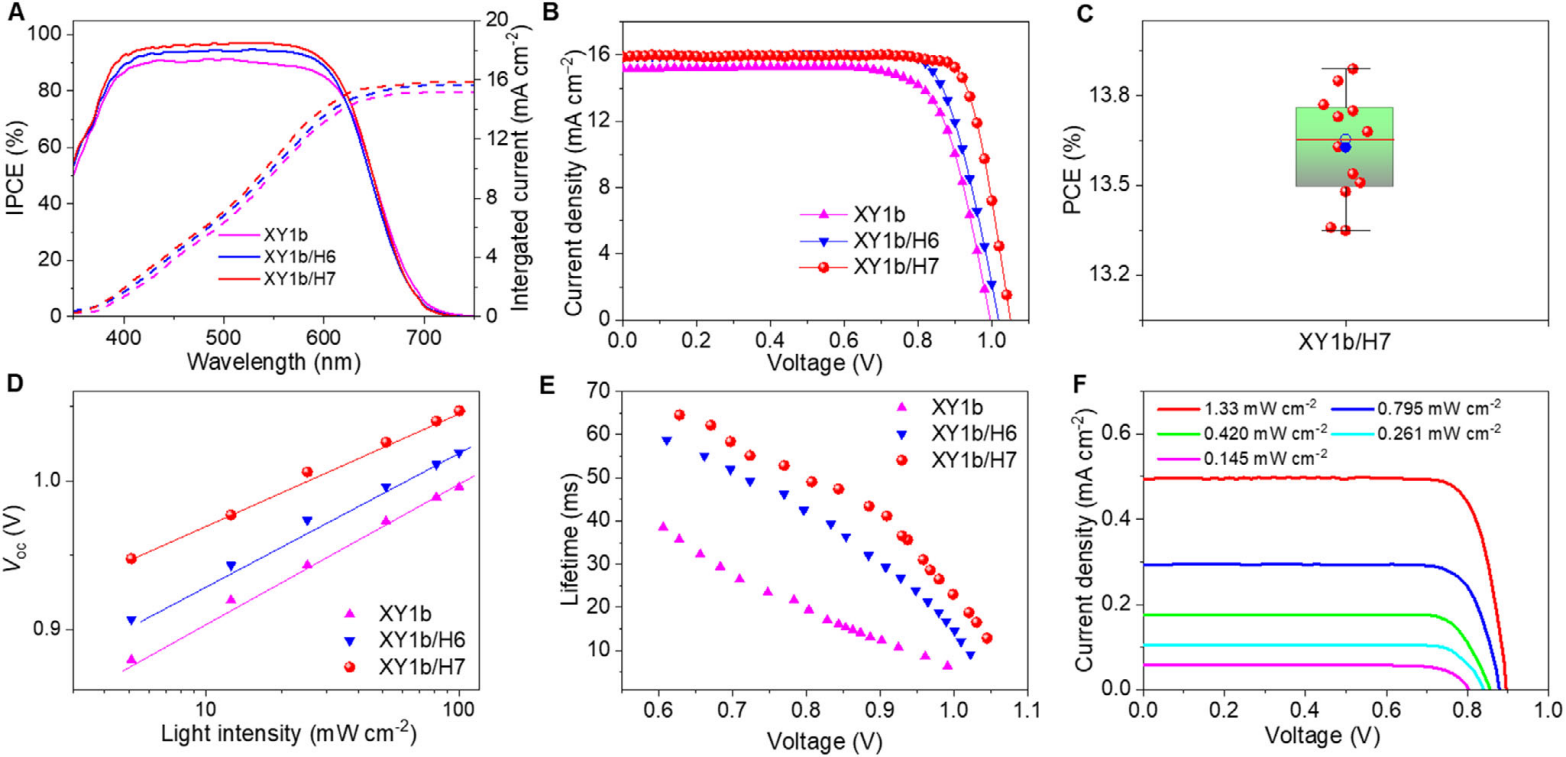
A) Plots of incident photon‐to‐electron conversion efficiency (IPCE) and integrated current density as a function of wavelength. B) Current−voltage (*J*−*V*) curves at an irradiance of 100 mW cm^−2^, AM1.5G conditions. C) Statistics of PCE of the co‐sensitized solar cells based on 12 samples of XY1b/**H7** device. D) Open circuit photovoltage (*V*_oc_) as a function of a series of light intensities (*I*) of the device based on XY1b and co‐sensitized devices, and the calculation of ideality factor (*n*). E) Comparison of electron lifetimes measured with the small‐pulse disturbance transient photovoltage decay method against voltage. F) *J–V* curves for XY1b/**H7** device recorded at different illuminated LED light intensities.

Because of the high *V*_oc_ attained and strong capability of restraining interfacial charge recombination, both **H6** and **H7** are good candidates to prepare co‐sensitized high‐efficiency devices in combination with narrow‐energy‐gap dye. Co‐sensitization of a panchromatic dye with a narrow‐spectral response dye co‐grafted onto the TiO_2_ surface can simultaneously enhance photocurrent and photovoltage, resulting in enhanced overall device performance. The panchromatic dye broadens light absorption across the visible spectrum, while the wide‐energy‐gap dye can suppress charge recombination and optimize dye packing, thereby contributing to higher efficiency.^[^ ^35^, ^36^, ^50^, ^51^, ^52^, ^53^, ^54^ ^]^ Herein, we selected the state‐of‐the‐art dye XY1b to fabricate Cu‐tmby based DSCs with **H6** and **H7**. As shown in Figure S5 (Supporting Information), the **H6** or **H7** titania films have intense absorption ≈350–500 nm, compensating for the weak absorption of XY1b within the blue and green domain, allowing for efficient sunlight absorption between 400 and 700 nm. The sensitized titania films also exhibit a similar result. The IPCE spectrum of the co‐sensitized DSC displays higher peak values of over 90% than that of XY1b‐based cells. (**Figure** **5**A) As Figure 5B shows, the XY1b alone cell exhibits a *J*_sc_ of 15.16 mA cm^−2^, a *V*_oc_ of 996 mV, and FF of 75.1%, yielding a PCE of 11.3%. Compared to the XY1b cell, the co‐sensitized cell XY1b/**H6** exhibits a significantly augmented *J*_sc_ of 15.90 mA cm^−2^, an enhanced *V*_oc_ of 1019 mV, an augmented FF of 78.5%, affording a remarkably improved PCE of 12.7%. On the other hand, the XY1b/**H7** cell acquires a good *J*_sc_ of 15.89 mA cm^−2^, a *V*_oc_ of 1051 mV, and a highest FF of 82.1%, yielding an impressive PCE of 13.7% (Table 2). The *J−V* curves of the best device based on XY1b/**H7** recorded at different scan directions are shown in Figure S7 (Supporting Information). There is no obvious hysterisis in our device. Figure 5C and Figure S6 (Supporting Information) show a set of statistical results obtained from 12 individual solar cells, and the device parameters demonstrate the good performance reproducibility.

For further exploring the synergistic effect of the co‐sensitization strategy, we recorded the *J‐V* curves under a series of light irradiance. We fitted the *V*_oc_ dependence on the light intensity (*I*) to evaluate the effect on non‐radiative recombination. The ideality factor (*n*) can be acquired via Equation (1):

(1) $$n=\frac{q}{k_{B}T}\frac{dVoc}{dlnI}$$

where *q* is the elementary charge, *k*_B_ is the constant of Boltzmann, T is the room temperature 298 K, and *I* is the incident light density (mW cm^−2^). *n* relates to an ideal (*n* = 1) and non‐ideal (*n* > 1) diode. The direct band‐to‐band radiative charge recombination identifies *n* ≈ 1 for highly efficient nanocrystal‐based solar cells. *n* usually deviates from unity for organic solar cells due to different recombination pathways in inter‐band transitions, such as trap‐assisted charge recombination. As shown in Figure 5D, concerning XY1b counterpart (*n* = 1.46), the co‐sensitized DSCs have the smaller value of *n*, being 1.28 for XY1b/**H7**, and 1.40 for XY1b/**H6**. The low value of *n* of co‐sensitized solar cells suggests that the co‐sensitized strategy largely restrains interfacial charge recombination from trap states. As the charge extraction (CE) measurements are shown in Figure S8 (Supporting Information), it is detected that the co‐sensitized device based on XY1b/**H7** possesses higher charge density, agreeing with the higher photocurrent obtained from the *J−V* measurements. At a given density of extracted charge, the electron lifetimes of co‐sensitized devices are longer than that of the XY1b counterpart (Figure 5E). As mentioned above, both **H6** and **H7** possess a strong ability to reduce interfacial charge recombination. Therefore, the combination of any of these molecules with XY1b results in a decrease of the recombination. Together with the increase in the photocurrent density, there is a consequent increase in performance in both cases.

### 2.6 Photovoltaic Device under Ambient LED Illumination

With the development of the global digital economy, the Internet of Things (IoT) ecosystem is gradually growing. Indoor photovoltaics are promising alternatives to traditional batteries to support low‐power IoT devices. It is reported that DSCs can work highly efficiently in ambient light, with an efficiency of over 30%.^[^ ^26^, ^37^, ^38^ ^]^ We tested the device performance of the highly efficient co‐sensitized XY1b/**H7** cell by using 18 NR‐T TiO_2_ electrodes, under light‐emitting diode (LED) lamp illuminance, simulating the ambient light. Solar cells tested under the LED light employed an electrolyte composed of 0.08 m [Cu(I)(tmby)_2_]TFSI and 0.04 m [Cu(II)(tmby)_2_](TFSI)_2_ complexes together with 0.1 m LiTFSI and 0.6 m NMB in 3‐methoxypropionitrile. The less volatile and low toxic 3‐methoxypropionitrile employed here is well‐suited for their long‐term durability and commercial applications. The setup of LED‐light PV measurements is shown in Figure S9 (Supporting Information). Figure 5F shows its corresponding *J–V* curves, and Table S2 (Supporting Information) lists the photovoltaic performance parameters for the co‐sensitized XY1b/**H7** device under various illuminated intensities from the LED light source (500–4500 lux). The DSC operating under ambient light is less demanding concerning the mass transport limitation of the redox shuttles compared with intense irradiation, resulting in a high fill factor.^[^ ^32^ ^]^ At varying illumination intensities between 500 to 4500 lux, the co‐sensitized device exhibits high FFs of over 80% and remarkable PCEs of 26.2%–29.7%. When illuminated by LED lamp at the light intensity of 1500 lux, the XY1b/**H7** device achieves a *V*_oc_ of 857 mV, a *J*_sc_ of 0.175 mA cm^−2,^ and FF of 83.2%, yielding a power density of 0.1247 mW cm^−2^ and the corresponding outstanding PCE of 29.7%. For comparison purposes, we also used perovskite semiconductors (PSK) solar cells following the structure ITO/CP204/1.55 eV perovskite/PC61BM/BCP/Ag to test the performance under LED lamp illuminance. As presented in Figure S9 and Table S3 (Supporting Information), the PSK device shows a PCE of 24.9%, lower than the performance of co‐sensitized solar cells based on XY1b/**H7** at the light intensity of 1500 lux. The excellent PCE under LED light illuminance confers the co‐sensitized device to potentially practical indoor light applications **Table** S**2**.

**Table 2:** Photovoltaic parameters of DSCs measured under standard AM1.5G sunlight.

Dye	$J_{\mathrm{sc}}^{\mathrm{IPCE}}$ ^a)^[mA cm^−2^]	*J*_sc_[mA cm^−2^]	*V*_oc_[mV]	FF(%)	PCE(%)
**H6**	11.26 ± 0.11	11.40 ± 0.13	1151 ± 2	76.2 ± 0.2	10.0 ± 0.1
**H7**	11.04 ± 0.13	11.13 ± 0.12	1220 ± 3	72.5 ± 0.3	9.8 ± 0.1
**XY1b**	15.20 ± 0.18	15.16 ± 0.17	996 ± 3	75.1 ± 0.4	11.3 ± 0.2
**XY1b/H6**	15.78 ± 0.15	15.90 ± 0.14	1019 ± 3	78.5 ± 0.2	12.7 ± 0.3
**XY1b/H7**	15.83 ± 0.16	15.89 ± 0.15	1051 ± 2	82.1 ± 0.2	13.7 ± 0.2

^a)^ $J_{\mathrm{sc}}^{\mathrm{IPCE}}$ was computed by wavelength integral of the samples of the IPCE measured at the short‐circuit and the standard AM 1.5G emisssion specturum. (BenWin+).

## 3 Conclusion

In summary, we designed and synthesized two D‐π‐A organic dyes, **H6** and **H7,** composed of a Hagfeldt donor with n‐hexyl and n‐dodecyl chains. These are combined with bis‐hexylthiophene as a π linker and the electron acceptor 4‐(benzo[c][1,2,5]thiadiazol‐4‐yl)benzoic acid (BTBA). The **H7** cell, in combination with a copper electrolyte, produces a high photovoltage of 1.22 V, owing to retarded interfacial charge recombination, a longer excited state emission lifetime, and excellent light‐harvesting capabilities. The co‐sensitization of XY1b and **H7** results in highly efficient dye‐sensitized solar cells (DSCs) with an impressive power conversion efficiency (PCE) of 13.7% under standard AM1.5G sunlight conditions and a remarkable PCE of 29.7% under LED light illumination. Our work highlights the significance of careful molecular engineering of high‐photovoltage‐output co‐sensitizers to enhance the photovoltaic performance of DSCs utilizing copper redox electrolyte through the tunning of the excited state emission lifetime and the light harvesting properties.

## 4 Experimental Section

### Fabrication of Solar Cells

The mesoporous TiO_2_ electrodes were fabricated following the literature procedure.^[^ ^37^ ^]^ The fluorine‐doped tin oxide (FTO) glass (Nippon Sheet Glass, NSG, 10 ohms sheet resistance) was sequentially thoroughly cleaned with water, acetone, and ethanol. After drying with compressed air, the FTO glass was further cleaned by UV‐ozone for 20 min. A compact TiO_2_ (c‐TiO_2_) layer was deposited on top of the FTO glass by spray pyrolysis with O_2_ as the carrier gas as follows: the FTO substrate was first preheated to 450 °C followed by spraying a precursor solution of titanium diisopropoxide bis(acetylacetonate) (75 wt.% in isopropanol) that had been diluted with isopropanol by a volume ratio of 1:9 and the addition of 4% volume ratio of acetyl acetone. After spray pyrolysis, the FTO/c‐TiO_2_ substrate was stored at 450 °C for 1 h before cooling to room temperature. Using a screen printer, the TiO_2_ pastes of 30 NR‐D (Greatcell Solar Limited) and the light‐scattering TiO_2_ particles (Greatcell Solar Limited, 18NR‐AO) were deposited on the spraying FTO glass. The TiO_2_ films were patterned in a square area of 0.16 cm^2^. The mesoporous TiO_2_ films composed of ≈4.0 µm transparent layer and ≈4.0 µm light‐scattering layer were obtained after the films were sintered at 500 °C using temperature programmed and gradually cooled to room temperature. The mesoporous TiO_2_ films were further treated with 20 mM TiCl_4_ solution for 45 min in an oven at 70 °C. Those type of TiO_2_ electrodes were used for fabricating solar cells operating under AM1.5G sunlight. Another type of electrode was made by screen printing the transparent layer of TiO_2_ film with TiO_2_ pastes 18 NR‐T (Greatcell Solar Limited) and the light‐scattering layer of TiO_2_ particles (Greatcell Solar Limited, 18NR‐AO). These types of electrodes were used for making solar cells operating under LED light. The mesoporous TiO_2_ electrodes were stained by immersing them into dye solutions at room temperature for a fixed time before sintering at 500 °C in air for 30 min and cool down to 90 °C. The dye solutions of H6 and H7 were prepared by dissolving 0.1 mm of the corresponding dye in ethanol, iso‐propanol, respectively. The dye solution of XY1b was made by dissolving 0.1 mm XY1b and 0.5 mM chenodeoxycholic acid in CF/EtOH (*v*/*v*, 1/9). The solution for co‐sensitization of XY1b/H6 and XY1b/H7 was prepared by dissolving 0.1 mm XY1b, and 0.5 mm chenodeoxycholic acid, and 0.1 mM H6 or H7, in CF/EtOH (*v*/*v*, 1/9). The counter electrode (the PEDOT deposited onto FTO glass) was prepared using the electrical deposition technique following the literature protocol.^[^ ^55^ ^]^ Both electrodes were pressed together mechanically without a spacer and further sealed with UV light curing glue (UV RESIN), which was quickly solidified by UV light (Alonefire SV41 UV Flashlight). The electrolyte was injected into the sealed electrodes through a predrilled hole on the counter electrode to complete the fabrication of the sandwich‐type DSC. The hole was sealed with the above UV light‐curing glue. All the devices for characterizations were hermetically sealed. The copper‐based electrolyte consisting of 0.16 m [Cu(I)(tmby)_2_]TFSI and 0.08 m [Cu(II)(tmby)_2_](TFSI)_2_ complexes with 0.1 M LiTFSI and 0.6 M NMB in acetonitrile was used to obtain high efficiency under AM1.5G sunlight. Device tested under the LED light employed an electrolyte composed of 0.08 m [Cu(I)(tmby)_2_]TFSI and 0.04 m [Cu(II)(tmby)_2_](TFSI)_2_ complexes together with 0.1 m LiTFSI and 0.6 m NMB in 3‐methoxypropionitrile.

### Electrochemical Characterization

The electrochemical characterization of metal oxide surface‐attached dyes was conducted using a BioLogic SP150e potentiostat in a classical three‐electrode configuration with electrochemical cyclic voltammetry (CV) procedures. A dye adsorbed on a 4 µm thick transparent mesoscopic TiO_2_ film served as the working electrode, while Ag/AgCl and platinum wire acted as the reference and counter electrodes, respectively. A 0.1 m Tetrabutylammonium hexafluorophosphate (TBAPF_6_) solution was utilized as the supporting electrolyte, and the scan rate was set to 50 mV s^−1^. The ferrocene/ferrocenium redox system functioned as an internal standard to calibrate the quasi‐reference electrode before and after measurements.

### Photophysical Measurements and Data Analysis

The static UV–Vis spectra were analyzed using the UV‐2401PC spectrometer (Agilent). The steady fluorescence spectra were recorded with the LifeSpec II (Edinburgh). Femtosecond fluorescence traces were captured with a time‐resolved fluorescence spectrometer (Halcyone Fire, Ultrafast). The fundamental pulses were produced by a Ti: Sapphire laser system (Astrella, 800 nm, 35 fs, 7 mJ/pulse, 1 kHz repetition rate, Coherent Inc.). The output of the femtosecond pulses at 800 nm was divided into two parts: a larger portion was directed to one optical parametric amplifier (OPA) to generate the pump pulse (530 nm), while a smaller portion was sent to another OPA to create the gate pulse (1200 nm). The wavelength‐dependent fluorescence emitted from the randomly moving sample and the 1200 nm gate pulse were focused onto a BBO crystal to produce a sum frequency light, which then passed through a monochromator and was detected by a photomultiplier tube (PMT). Sub‐nanosecond transient absorption (TA) traces were recorded using a pump‐probe transient absorption spectrometer (EOS, Ultrafast System). The time‐dependent PL intensities at all detected wavelengths of each dye were globally fitted with four times constants according to Equation (2)

(2) $$I_{PL}\propto \sum_{i=1}^{4}A_{i}\exp\left(\frac{-t}{\tau_{i}}\right)⊗\mathrm{IRF},$$

where *t* and $\bar{\tau }$ represent the delay time and the time constant, respectively. The amplitude‐weighted average lifetimes ($\bar{\tau }$) of PL decays can be derived from Equation (3),

(3) $$\bar{\tau }=\sum_{i=1}^{n}Ai\tau i/\sum_{i=1}^{n}Ai\left(Ai>0\right)$$

The calculation of quenching yield is based on Equation (6),

(4) $$QY\left(\lambda \right)=\left(1-\frac{\bar{\tau }\left(T,\lambda \right)}{\bar{\tau }\left(A,\lambda \right)}\right)\times 100\%$$

The transient absorption traces at 1300 nm were fitted with a multi‐exponential function for easy determination of the half‐reaction time constants. The calculation of hole injection yield is based on Equation (5),

(5) $$\phi_{\mathrm{hi}}=\left(1-\frac{t_{1/2}^{\mathrm{Cu}}}{t_{1/2}^{\mathrm{inert}}}\right)\times 100\%.$$

Normalized transient absorption traces upon femtosecond laser pulse excitation of H6 and H7‐grafted titania films soaked with an inert electrolyte composed of 0.6 m *N‐*methylbenzimidazole and 0.1 m lithium bis(trifluoromethanesulfonyl)imide in acetonitrile and a Cu‐tmby electrolyte, respectively. Excitation wavelength: 525 nm for H6 and H7. Pulse fluence: 35 µJ cm^−2^. Probe wavelength: 1300 nm. The detailed procedure followed the literature.^[^ ^25^ ^]^

### Dye Loading Amount Measurements

The kinetics of dye loading were measured using the methodology outlined in the literature.^[^ ^27^ ^]^ A 12 mL fresh dye solution was divided into three equal parts (solutions 1, 2, and 3). An FTO glass covered with a 4.0‐µm‐thick transparent TiO_2_ film and a blank sheet of FTO glass of the same size were immersed in solutions 1 and 2, respectively. It was noted that the H6 and H7 dye solution were prepared by dissolving in ethanol, iso‐propanol, respectively. The dye‐soaking time was recorded, and then the TiO_2_ film was removed from solution 1 and washed with 1 g of acetonitrile to eliminate the weakly anchored dyes. The dye stock solution was mixed with acetonitrile to achieve a total weight of 5 g. The bare FTO glass in solution 2 was treated using the same method as the TiO_2_ film in solution 1. Additionally, 1 g of acetonitrile was also added to solution 3. Furthermore, 3 g of THF solvent was added to each vial of solutions 1, 2, and 3 to ensure the complete dissolution of the dyes. Finally, the UV–vis spectra of these three solutions were measured. The amount of dye loading, expressed as the number of adsorbed moles of dye per unit film area and unit film thickness (*c*_m_), was calculated using Equation (6):

(6) $$C_{m}=\frac{\left(Abs_{2}-Abs_{1}\right)\times c\times V}{Abs_{3}\times A\times d}$$

where Abs_1_, Abs_2,_ and Abs_3_ were the absorbance values of final solutions 1, 2, and 3 at the maximal absorption wavelength, respectively. *c* and *V* were the concentration and volume of fresh solutions 1, 2, and 3, respectively. *A* and *d* stand for the area and thickness of the TiO_2_ film, respectively.

### Device Characterization

The *J‐ V* curves were recorded using a solar simulator (ABET 11000) and a source meter (Keithley 2400). The curves were measured under 1 Sun conditions (100 mW cm^−2^, AM 1.5G) calibrated with a Si reference cell. The scan rate used was 0.125 V/s. The active area of the devices was 0.08 cm^2^. The IPCE was measured with BenWin+ Spectral Acquisition Software from BENTHAM. Photoinduced charge extraction (CE) and transient photovoltage (TPV) measurements were performed using a white LED controlled by a programmable power supply and a control box that switched between open‐ and short‐circuit states. All signals were recorded using a Yokogawa DLM2052 oscilloscope, which captured voltage drops. Light perturbation pulses for TPV were supplied by Oxxius lasers with a 520 nm wavelength.

## Conflict of Interest

The authors declare no conflict of interest.

## Supporting information



Supporting Information

Supporting Information

## Data Availability

The data that support the findings of this study are available in the supplementary material of this article.
