# Supplementary material for: Co‐Sensitized Solar Cell Achieves 13.7% Efficiency with Bis‐Hexylthiophene Dyes
Source: Adv Sci (Weinh). 2025 Aug 18;12(42):e09116. doi: 10.1002/advs.202509116 (PMC12622478; doi:10.1002/advs.202509116)
Supplement: Supplementary file 1 — Supporting Information [file ADVS-12-e09116-s001.docx]

**Supporting information**

**Co-Sensitized Solar Cell Achieves 13.7% Efficiency with Bis-Hexylthiophene Dyes**

Heng Wu, Laia Marín Moncusí, Jing Li, Eugenia Martínez-Ferrero, Peng Wang, and Emilio Palomares*

**Materials.** Lithium bis(trifluoromethanesulfonyl)imide (LiTFSI), *N*-methylbenzimidazole (NMB), 1-ethyl-3-methylimidazolium bis(trifluoromethanesulfonyl)imide (EMITFSI), ferrocene (Fc), bis(pinacolato)diboron, tetrabutylammonium hexafluorophosphate (TBAPF_6_), *N*-Bromosuccinimide (NBS), [1,1'-bis(diphenylphosphino)ferrocene]dichloropalladium(II) (Pd(dppf)Cl_2_), palladium(II) acetate (Pd(OAc)_2_), 2-(2,6-dimethoxybiphenyl)-dicyclohexylphosphine (Sphos), tricyclohexylphosphine tetrafluoroborate (PCy_3_·HBF_4_), pivalic acid (PivOH), potassium carbonate (K_2_CO_3_), potassium acetate, (KOAc), potassium hydroxide (KOH), potassium phosphate (K_3_PO_4_), 3-hexylthiophene, hydrochloric acid, and chenodeoxycholic acid, were purchased from Sigma-Aldrich. Toluene, chloroform, dioxane, and tetrahydrofuran (THF), 3-methoxypropionitrile were dried and distilled before use. The powders of [Cu(I)(tmby)_2_][TFSI], [Cu(II)(tmby)_2_][TFSI]_2_, and XY1b were purchased from Dyenamo AB company, and used as received without further purification. 2-bromo-3-hexylthiophene^[1]^ (2), 3,3'-dihexyl-2,2'-bithiophene^[1]^ (**3**), ethyl 4-(7-bromobenzo[c][1,2,5]thiadiazol-4-yl)benzoate^[2]^, *N*-(2',4'-bis(hexyloxy)-[1,1'-biphenyl]-4-yl)-2',4'-bis(hexyloxy)-*N*-(4-(4,4,5,5-tetramethyl-1,3,2-dioxaborolan-2-yl)phenyl)-[1,1'-biphenyl]-4-amine^[3]^ (**6**), and *N*-(2',4'-bis(dodecyloxy)-[1,1'-biphenyl]-4-yl)-2',4'-bis(dodecyloxy)-*N*-(4-(4,4,5,5-tetramethyl-1,3,2-dioxaborolan-2-yl)phenyl)-[1,1'-biphenyl]-4-amine^[3]^ (**7**) were synthesized according to the respective literature procedures. Other chemical agents were purchased and used without further purification.

**Detailed Synthetic Procedures and Characterization Data**. The photosensitizing dyes **H6** and **H7** were prepared according to **Scheme S1**.

**Scheme S1** Synthetic procedures of dyes **H6** and **H7**.

Synthesis of 3,3'-dihexyl-2,2'-bithiophene (**3**).

Bromination of 3-hexylthiophene (**1**) with NBS obtained 2-bromo-3-hexylthiophene (**2**) under mixed solvent chloroform and acetic acid. Bis(triphenylphosphine)nickel(II) dichloride (1.3 g, 2 mmol), triphenylphosphine (1.0 g, 4 mmol), zinc powder (1.3 g, 20 mmol), and KI (80 mg, 0.5 mmol) were added into anhydrous THF under nitrogen atmosphere. The mixture was further stirred at room temperature for 0.5 h. The colour became reddish brown. Then, **2** (4.94 g, 20 mmol) was added. The mixture was refluxed overnight and then cooled down. The mixture was extracted with ethyl acetate three times. The crude product was purified by column chromatography with hexane as the eluent. Pure product **3** as colourless oil was afforded (2.3 g, 89%). H NMR (400 MHz, CDCl_3_) δ 7.31 (s, 2H), 6.99 (d, *J* = 5.2 Hz, 2H), 2.56 – 2.49 (m, 4H), 1.58 – 1.53 (m, 4H), 1.32 – 1.25 (m, 12H), 0.89 (d, *J* = 6.9 Hz, 6H). ^1^H NMR results were consistent with the data in the literature.^[1]^

Synthesis of ethyl 4-(7-(3,3'-dihexyl-[2,2'-bithiophen]-5-yl)benzo[c][1,2,5]thiadiazol-4-yl)benzoate (**4**). In a dried Schlenk tube were dissolved **3** (442 mg, 1.3 mmol), ethyl 4-(7-bromobenzo[c][1,2,5]thiadiazol-4-yl)benzoate (480 mg, 1.3 mmol), K_2_CO_3_ (274 mg, 1.98 mmol) in toluene (10 mL). Then Pd(OAc)_2_ (15 mg, 66 μmol), PCy_3_·HBF_4_ (49 mg, 132 μmol), and PivOH (40 mg, 386 μmol) were added to the reaction mixture in a nitrogen-filled atmosphere, which was refluxed for 24 h. The mixture was extracted three times with chloroform before the organic phase was washed with water and dried over anhydrous sodium sulfate. After solvent removal under reduced pressure, the crude product was purified by column chromatography (dichloromethane/hexane, 1/2, *v*/*v*) on silica gel to yield a red solid as the desired product **4** (390 mg, 47.9% yield). ^1^H NMR (400 MHz, CDCl_3_) δ 8.30 – 8.19 (m, 2H), 8.11 (s, 1H), 8.10 – 8.05 (m, 2H), 7.93 (d, *J* = 7.5 Hz, 1H), 7.78 (d, *J* = 7.5 Hz, 1H), 7.36 (d, *J* = 5.2 Hz, 1H), 7.03 (d, *J* = 5.2 Hz, 1H), 4.46 (q, *J* = 7.1 Hz, 2H), 2.72 – 2.53 (m, 4H), 1.66 (dh, *J* = 15.2, 7.8 Hz, 4H), 1.46 (d, *J* = 14.3 Hz, 3H), 1.39 – 1.25 (m, 12H), 0.89 (dt, *J* = 11.2, 6.8 Hz, 6H). ^13^C NMR (101 MHz, CDCl_3_) δ 166.39, 153.79, 152.65, 143.53, 142.62, 141.56, 138.47, 131.32, 131.11, 130.06, 130.03, 129.81, 129.79, 129.07, 128.92, 128.73, 128.61, 128.30, 127.27, 127.13, 127.00, 125.65, 125.18, 77.38, 77.07, 76.75, 61.07, 31.68, 30.76, 29.20, 29.16, 29.11, 29.00, 22.63, 22.61, 14.40, 14.11, 14.10.

Synthesis of ethyl 4-(7-(5'-bromo-3,3'-dihexyl-[2,2'-bithiophen]-5-yl)benzo[c][1,2,5]thiadiazol-4-yl)benzoate (**5**). Compound **4** (0.25 g, 0.41 mmol) was dissolved in THF (20 mL), and the solution was cooled to 0 ^o^C using an ice salt bath. NBS (72 mg, 0.41 mmol) in THF (5 mL) was added to the reaction mixture dropwise. Then the resulting solution was stirred at 0 ^o^C for 0.5 h. Water was added to terminate the reaction and the mixture was extracted by CH_2_Cl_2_ (20 mL × 3). After the combined organic phase was dried over Na_2_SO_4_, the solvent was removed under reduced pressure and the residue was purified by silica gel column chromatography (toluene/hexane, *v*/*v*, 1/2) to give the product **5**. The crude product **5** was used to synthesized H6 and H7 without further purification.

*Synthesis of 4-(7-(5'-(4-(bis(2',4'-bis(hexyloxy)-[1,1'-biphenyl]-4-yl)amino)phenyl)-3,3'-dihexyl-[2,2'-bithiophen]-5-yl)benzo[c][1,2,5]thiadiazol-4-yl)benzoic acid (****H6****)*:

Compound **5** (300 mg, 0.43 mmol), Compound **6** (478 mg, 0.52 mmol), Pd(OAc)_2_ (4.9 mg, 0.022 mmol), Sphos (8.9 mg, 0.022 mmol), K_3_PO_4_ (275 mg, 1.3 mmol) and dioxane/H_2_O (10 mL, *v*/*v*, 5/1) were added to a three-neck round-bottom flask under argon. After the reaction mixture was refluxed for 5 h, the solution was cooled to room temperature, brine (10 mL) was added into the solution and the mixture was extracted by CH_2_Cl_2_ (20 mL × 3). After the combined organic phase was dried over Na_2_SO_4_, the solvent was removed under reduced pressure and the residue was purified by silica gel column chromatography (dichloromethane/hexane, *v*/*v*, 1/2) to give the desired ethyl ester (430 mg, 71%).

In a round-bottom flask were dissolved the desired carboxylic ester (420 mg, 0.30 mmol) and KOH (334 mg, 5.94 mmol) in a solvent mixture of THF/H_2_O (20 mL, 3/1, *v*/*v*). The reaction mixture was refluxed for 5 h and then cooled to room temperature. Chloroform was added before the organic phase was washed with 0.1 M hydrochloric acid and water in turn and then dried over anhydrous sodium sulfate. After solvent removal under reduced pressure, the crude product was purified by column chromatography (chloroform/methanol, 10/1, *v*/*v*) on silica gel to yield a black solid as the desired product **H6** (380 mg, 93%). ^1^H NMR (400 MHz, CDCl_3_) δ 8.32 (d, *J* = 11.3 Hz, 2H), 8.15 (d, *J* = 6.7 Hz, 3H), 7.98 (d, *J* = 7.4 Hz, 1H), 7.84 (d, *J* = 7.5 Hz, 1H), 7.59 – 7.42 (m, 6H), 7.31 (s, 2H), 7.24 – 7.13 (m, 7H), 6.57 (dq, *J* = 5.6, 2.4 Hz, 4H), 4.00 (dt, *J* = 9.8, 6.5 Hz, 8H), 2.67 (dt, *J* = 22.6, 7.8 Hz, 4H), 1.87 – 1.63 (m, 13H), 1.48 – 1.28 (m, 36H), 0.98 – 0.86 (m, 18H). ^13^C NMR (101 MHz, CDCl_3_) δ 159.57, 156.99, 153.80, 152.70, 145.60, 143.62, 138.34, 131.12, 130.85, 130.52, 130.24, 129.25, 128.83, 123.84, 123.71, 123.01, 105.35, 100.46, 77.34, 77.02, 76.71, 68.43, 68.14, 31.70, 31.63, 31.49, 30.78, 29.33, 29.22, 29.16, 29.10, 25.78, 22.64, 22.61, 14.11, 14.06. HR-MS *m*/*z* calcd. for (C_87_H_105_N_3_O_6_S_3_): 1387.7166. Found: 1387.7169. IR (*ν*_max_, cm^-1^) 3032, 2924, 2855, 1687, 1603, 1580, 1491, 1466, 1421, 1320, 1269, 1179, 1132, 1018, 1002, 895, 831, 770, 724, 515. Melting point: 125.3 – 126.2 °C.

*Synthesis of 4-(7-(5'-(4-(bis(2',4'-bis(dodecyloxy)-[1,1'-biphenyl]-4-yl)amino)phenyl)-3,3'-dihexyl-[2,2'-bithiophen]-5-yl)benzo[c][1,2,5]thiadiazol-4-yl)benzoic acid (****H7****):*

Compound **5** (250 mg, 0.36 mmol), compound **7** (679 mg, 0.52 mmol), Pd(OAc)_2_ (4.9 mg, 0.022 mmol), Sphos (8.9 mg, 0.022 mmol), K_3_PO_4_ (275 mg, 1.3 mmol) and dioxane/H_2_O (10 mL, *v*/*v*, 5/1) were added to a three-neck round-bottom flask under argon. After the reaction mixture was refluxed for 5 h, the solution was cooled to room temperature, brine (10 mL) was added to the solution, and the mixture was extracted by CH_2_Cl_2_ (20 mL × 3). After the combined organic phase was dried over Na_2_SO_4_, the solvent was removed under reduced pressure, and the residue was purified by silica gel column chromatography (dichloromethane/hexane, *v*/*v*, 1/2) to give the desired product (480 mg, 76%). In a round-bottom flask were dissolved the desired ethyl ester (480 mg, 0.30 mmol) and KOH (334 mg, 5.94 mmol) in a solvent mixture of THF/H_2_O (20 mL, *v*/*v*, 3/1). The reaction mixture was refluxed for 5 h and then cooled to room temperature. Chloroform was added before the organic phase was washed with 0.1 M hydrochloric acid and water in turn and then dried over anhydrous sodium sulfate. After solvent removal under reduced pressure, the crude product was purified by column chromatography (chloroform/methanol, 10/1, *v*/*v*) on silica gel to yield a black solid as the desired dye **H7** (430 mg, 91%). ^1^H NMR (400 MHz, CDCl_3_) δ 8.36 – 8.28 (m, 2H), 8.22 – 8.07 (m, 3H), 7.98 (d, *J* = 7.4 Hz, 1H), 7.84 (d, *J* = 7.5 Hz, 1H), 7.57 – 7.45 (m, 6H), 7.30 (d, *J* = 3.5 Hz, 2H), 7.20 (ddd, *J* = 9.9, 4.9, 2.5 Hz, 7H), 6.57 (dq, *J* = 5.7, 2.5 Hz, 4H), 4.00 (dt, *J* = 10.0, 6.5 Hz, 8H), 2.67 (dt, *J* = 23.0, 7.8 Hz, 4H), 1.89 – 1.74 (m, 9H), 1.74 – 1.62 (m, 4H), 1.30 (dd, *J* = 16.2, 10.1 Hz, 85H), 0.95 – 0.85 (m, 18H). ^13^C NMR (101 MHz, CDCl_3_) δ 159.56, 156.98, 152.70, 145.61, 143.61, 133.19, 130.84, 130.51, 130.23, 129.25, 127.41, 126.30, 123.83, 122.99, 105.35, 100.48, 77.34, 77.22, 77.02, 76.70, 68.43, 68.14, 31.93, 31.92, 31.70, 30.77, 29.69, 29.65, 29.63, 29.61, 29.44, 29.37, 29.32, 29.22, 29.17, 29.13, 26.11, 22.70, 22.63, 14.13, 14.11. HR-MS *m*/*z* calcd. for (C_111_H_153_N_3_O_6_S_3_): 1721.0955. Found: 1721.0953. IR (*ν*_max_, cm^-1^) 3032, 2921, 2851, 1687, 1605, 1580, 1492, 1466, 1422, 1321, 1270, 1181, 1133, 1044, 1002, 895, 832, 771, 721, 537. Melting point: 77.4 – 78.5 °C.

**References**

[1] S. Yagai, M. Suzuki, X. Lin, M. Gushiken, T. Noguchi, T. Karatsu, A. Kitamura, A. Saeki, S. Seki, Y. Kikkawa, Y. Tani, K. Nakayama, Supramolecular engineering of oligothiophene nanorods without insulators: hierarchical association of rosettes and photovoltaic properties. *Chem. Eur. J.* **2014**, *20,* 16128.

[2] M. Zhang, Y. Wang, M. Xu, W. Ma, R. Li, P. Wang, Design of high-efficiency organic dye for titania solar cells based on the chromophoric core of cyclopentadithiophene-benzothiadiazole. *Energy Environ. Sci.* **2013**, *6*, 2944.

[3] D. Zhang, M. Stojanovic, Y. Ren, Y. Cao, F.T. Eickemeyer, E. Socie, N. Vlachopoulos, J.E. Moser, S. M. Zakeeruddin, A. Hagfeld, M. Grätzel, A molecular photosensitizer achieves a *V*_oc_ of 1.24 V enabling highly efficient and stable dye-sensitized solar cells with copper(II/I)-based electrolyte. *Nat. Commun*. **2021**, *12*, 1777.

**Figure S1.** Chemical structure of Narrowed energy gap photosensitizers XY1b, and co-sensitizer MS5.

**Figure S2.** UV-vis absorption spectra of **H6**, **H7**, and MS5 dissolved in THF.

**Figure S3.** Time-resolved photoluminescence decay traces of **H6** and **H7** titania films. Excitation wavelength: 405 nm.

**Table S1.** Time constants and amplitudes employed to fit fluorescence decays of dye grafted alumina and titania films^[a]^

|  | H6@ Al_2_O_3_ | H6@ TiO_2_ | H7 @ Al_2_O_3_ | H7@ TiO_2_ |
| --- | --- | --- | --- | --- |
| *τ*_1_ (ps) | 23 | 1.8 | 37 | 2.6 |
| *τ*_2_ (ps) | 194 | 5.7 | 348 | 7.3 |
| *τ*_3_ (ps) | / | 40 | / | 54 |
| *A*_1_ | 0.40 | 0.52 | 0.46 | 0.61 |
| *A*_2_ | 0.60 | 0.39 | 0.54 | 0.28 |
| *A*_3_ | / | 0.09 | / | 0.11 |
| *τ* (ps) | 126 | 6.8 | 205 | 9.5 |

[a] The amplitude-averaged lifetime of fluorescence at a certain wavelength was calculated with the equation 3. Excitation wavelength: 490 nm.

**Figure S4.** Plots of open-circuit photovoltages (*V*_oc_) as a function of short-circuit photocurrents (*J*_sc_).

**Figure S5.** UV-vis absorption spectra of **H6**, **H7**, XY1b, co-sensitized XY1b/**H6**, and XY1b/**H7** grafted at 4-μm-thick semi-transparent TiO_2_ film.


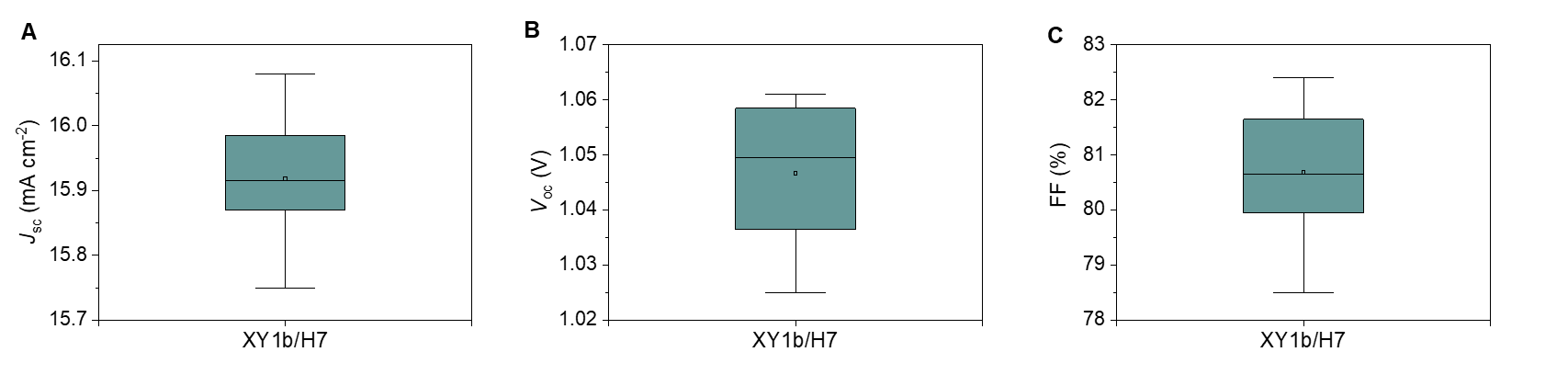


**Figure S6.** Device parameters *J*_SC_, *V*_oc_, FF, statistics with the co-sensitized solar cell based on XY1b/**H7**.

**Figure S7.** *J–V* curves of co-sensitized solar cells XY1b/**H7** recorded at different scan direction.

**Figure S8.** Charge density from the conduction band of dye-grafted titania films as a function of voltage for the DSCs.


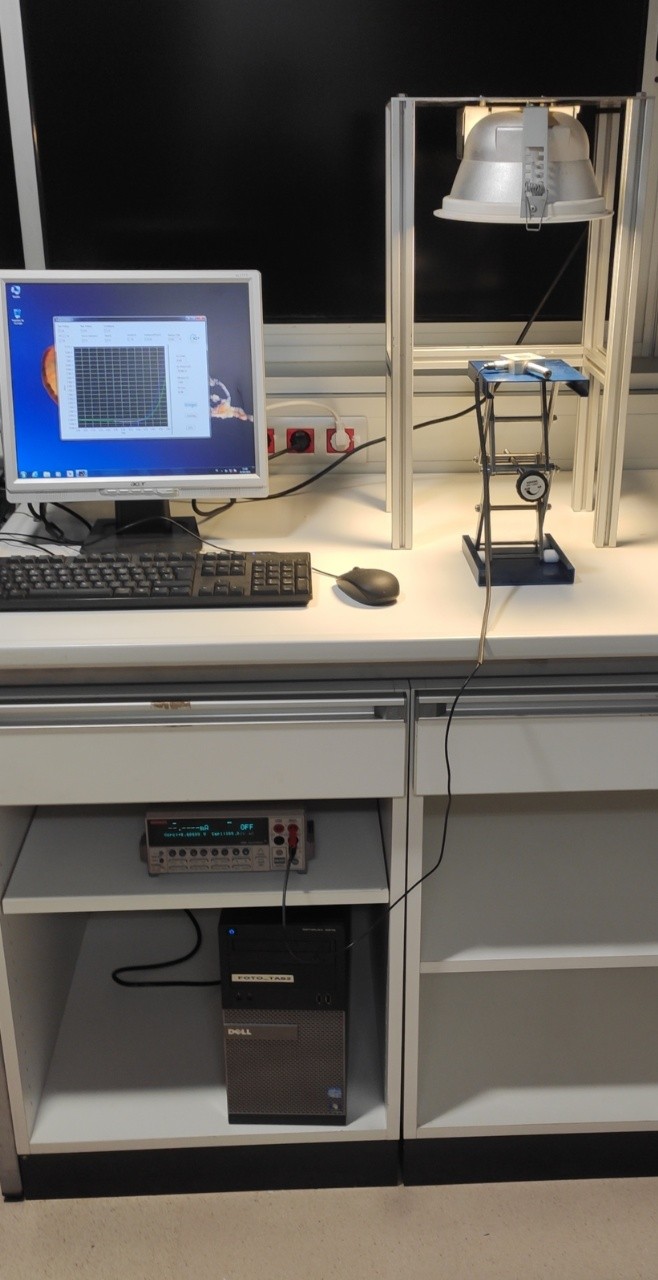


**Figure S9.** The LED-light PV measurements system. Indoor light was provided by the PHILIPS Master PL-C tube of 18W, the *J–V* curves were recorded at a scan rate of 0.02 V/s.

**Table S2.** Photovoltaic parameters of device incorporating XY1b with **H7** measured at a series of illumination intensity by using LED light

^[a]^PCE calculated from equation: PCE = P_out_ / P_in_.

**Figure S10.** *J–V* curves for PVK device recorded at illuminated LED light.

**Table S3.** Photovoltaic parameters of PSK device measured at a LED light illumination

**Appendix: ^1^H NMR, ^13^C NMR, and Mass Spectra, and ATR-FTIR Spectra of New Compounds**


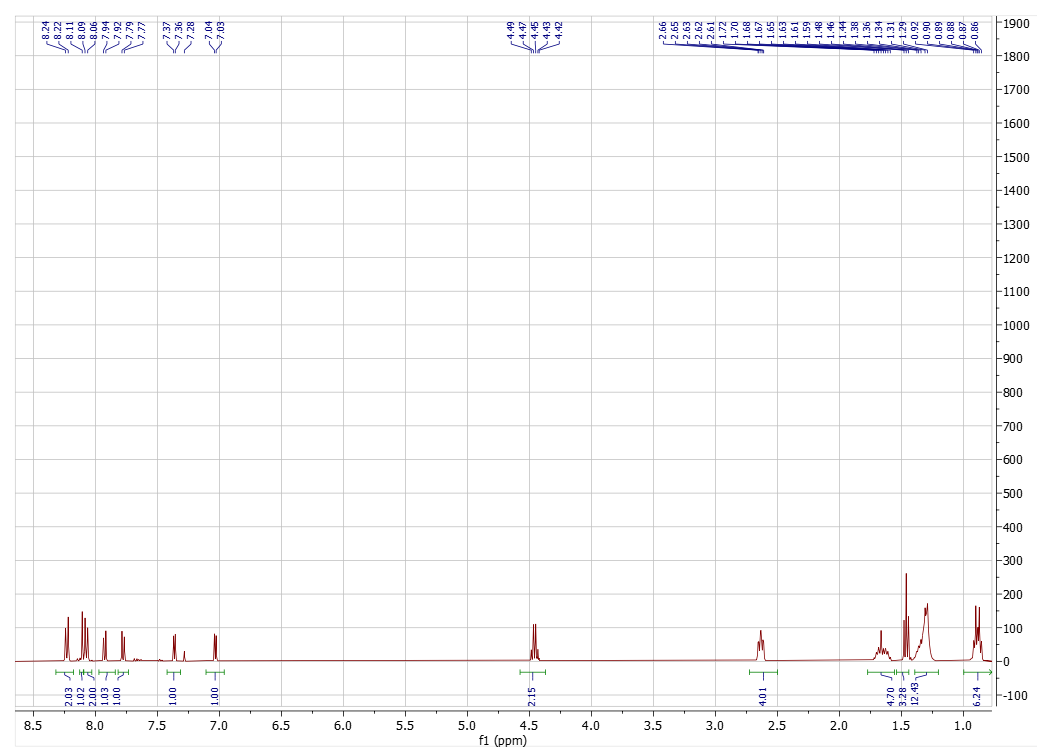


**Figure S11.** The ^1^H NMR (400 MHz) spectrum of compound **3** in CDCl_3_.


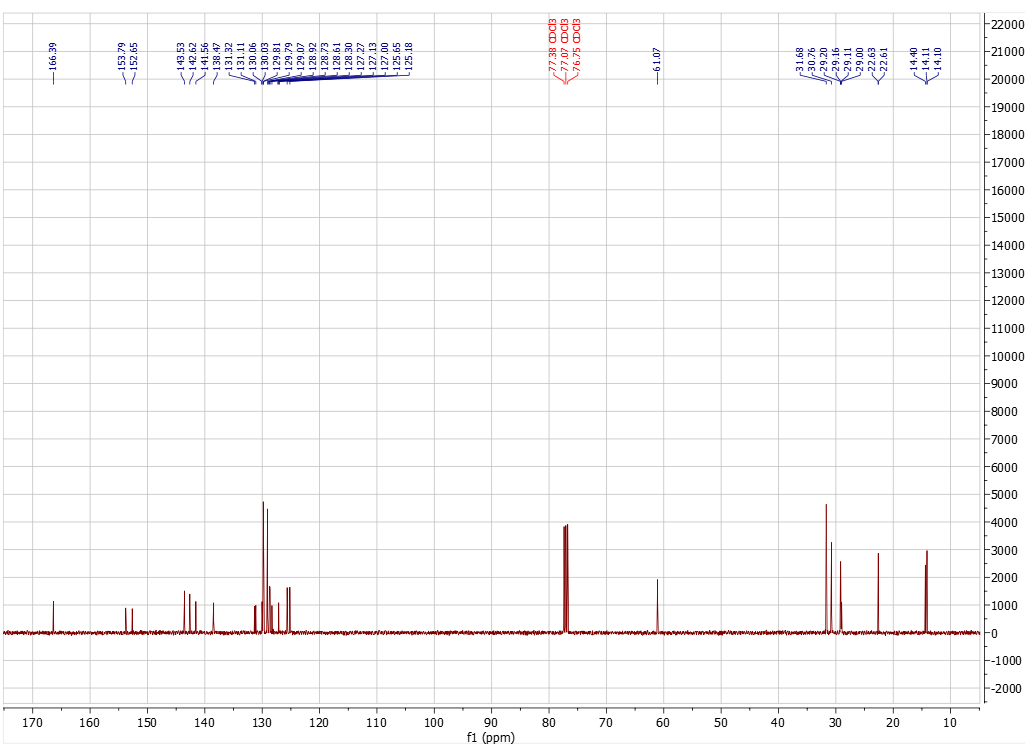


**Figure S12.** The ^13^C NMR (101 MHz) spectrum of compound **3** in CDCl_3_.


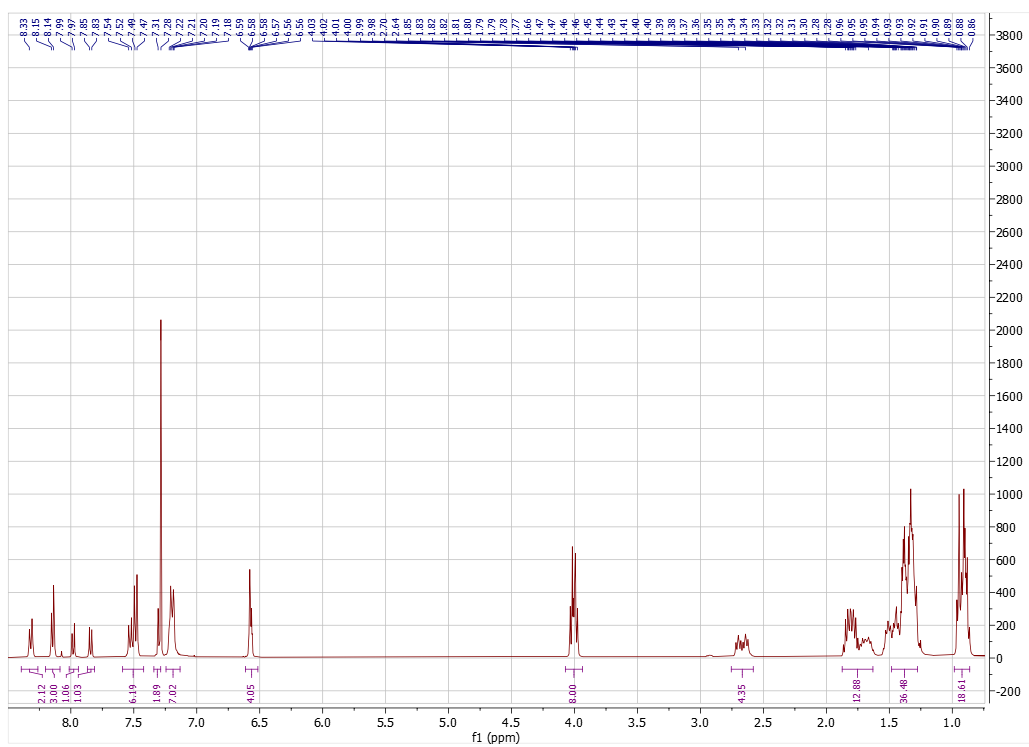


**Figure S13.** The ^1^H NMR (400 MHz) spectrum of compound **H6** in CDCl_3_.


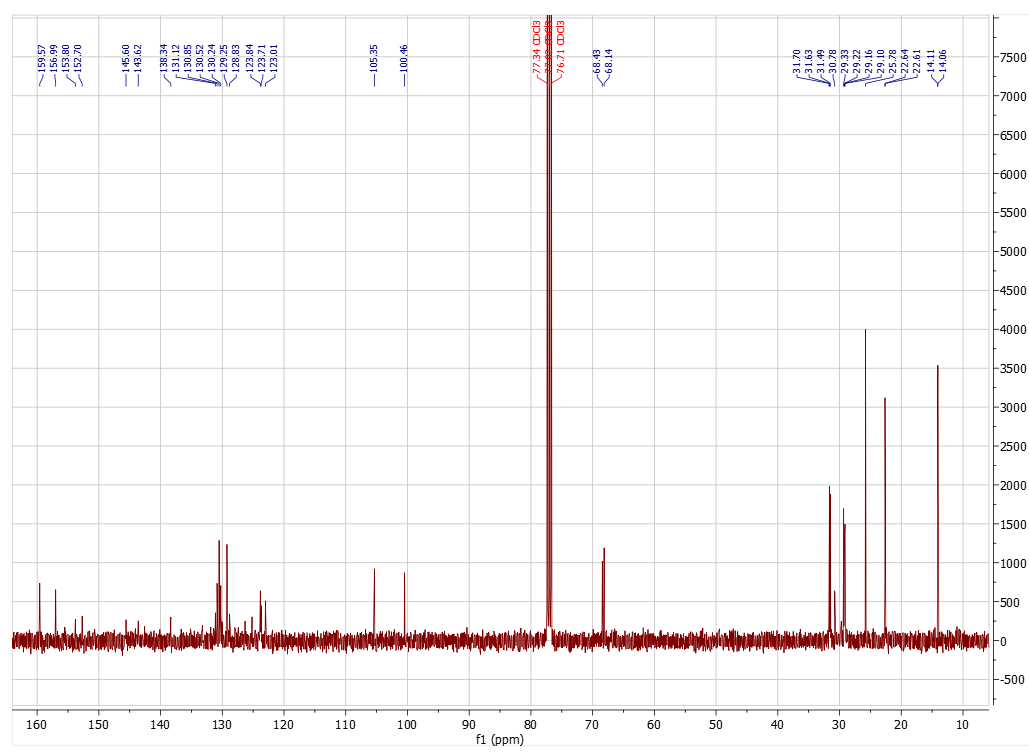


**Figure S14.** The ^1^C NMR (101 MHz) spectrum of compound **H6** in CDCl_3_.


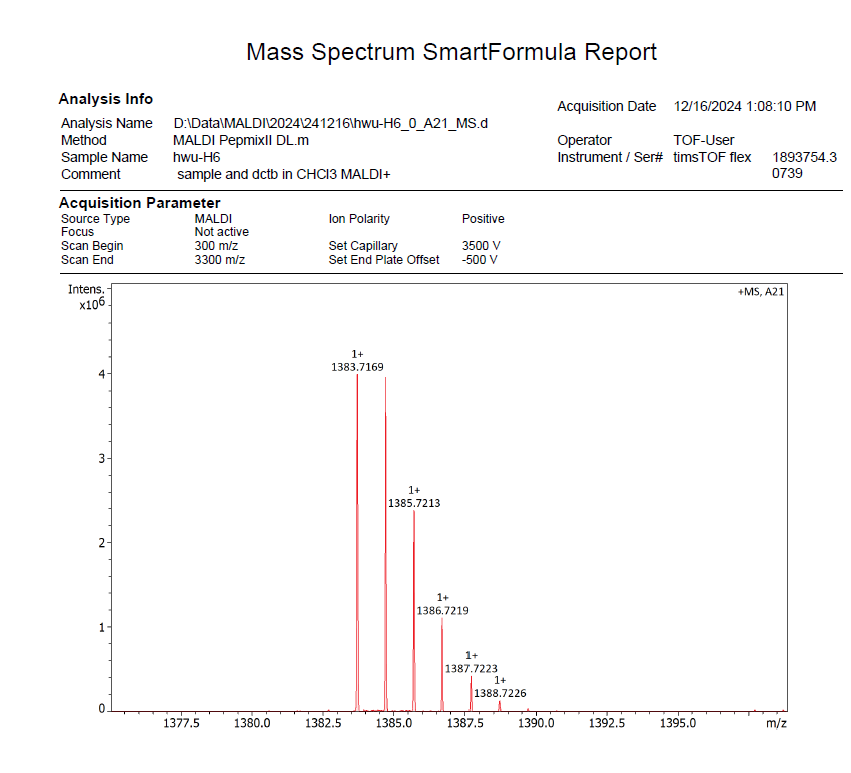


**Figure S15.** High resolution mass spectrum of **H6**.

**Figure S16.** ATR-FTIR spectrum of **H6**.


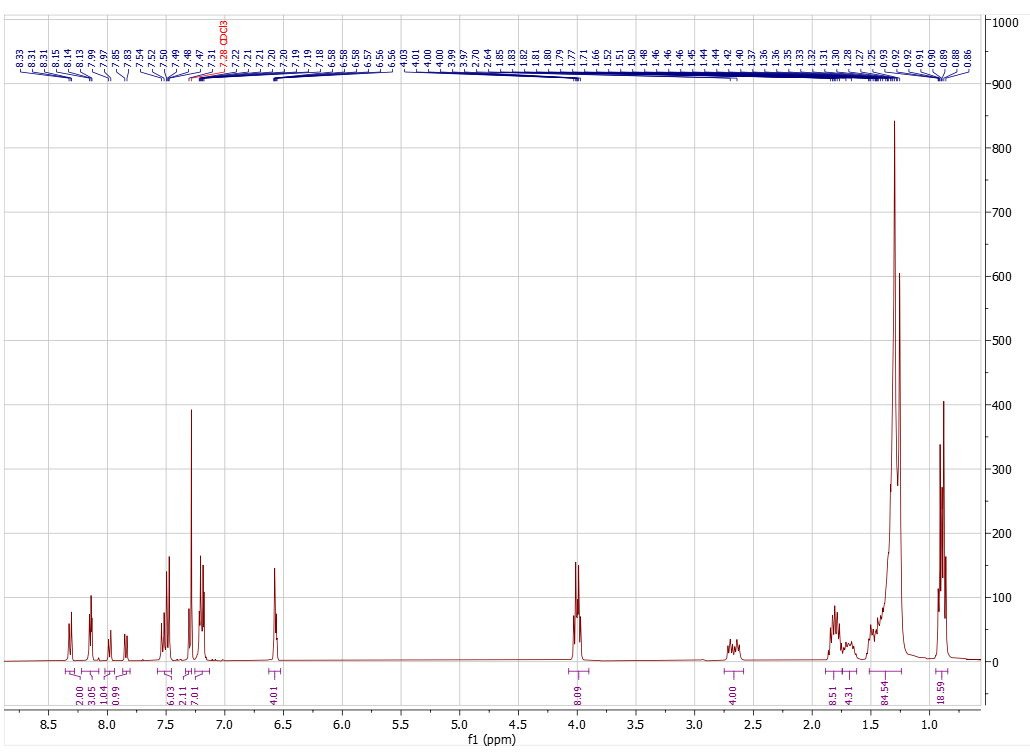


**Figure S17.** The ^1^H NMR (400 MHz) spectrum of compound **H7** in CDCl_3_.


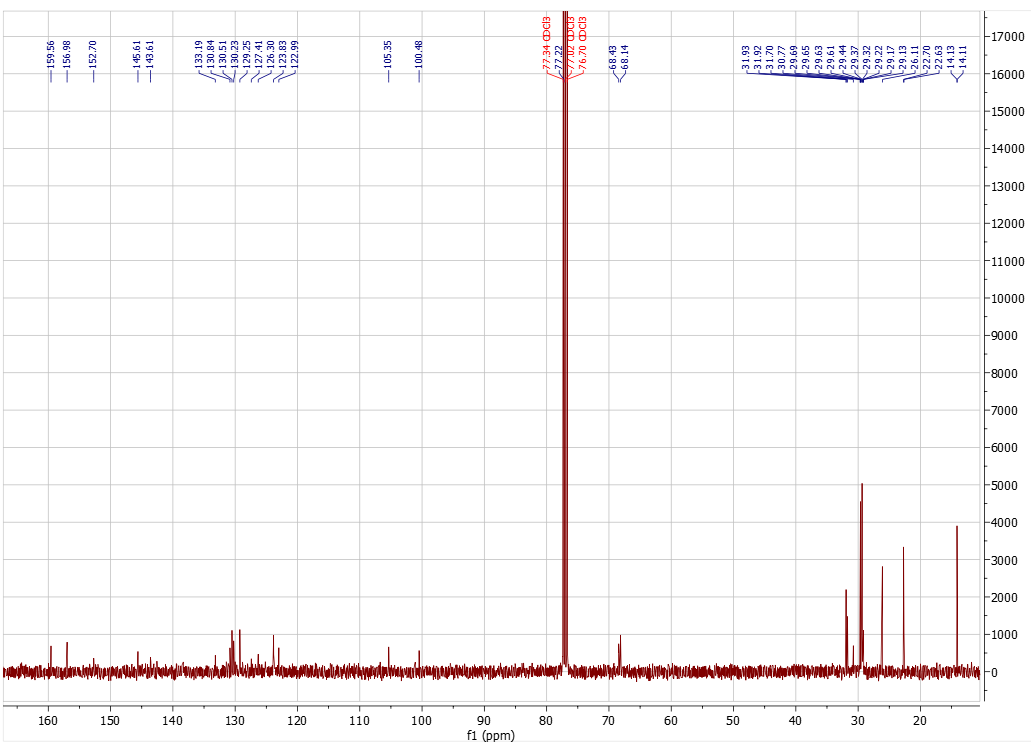


**Figure S18.** The ^1^C NMR (101 MHz) spectrum of compound **H7** in CDCl_3_.


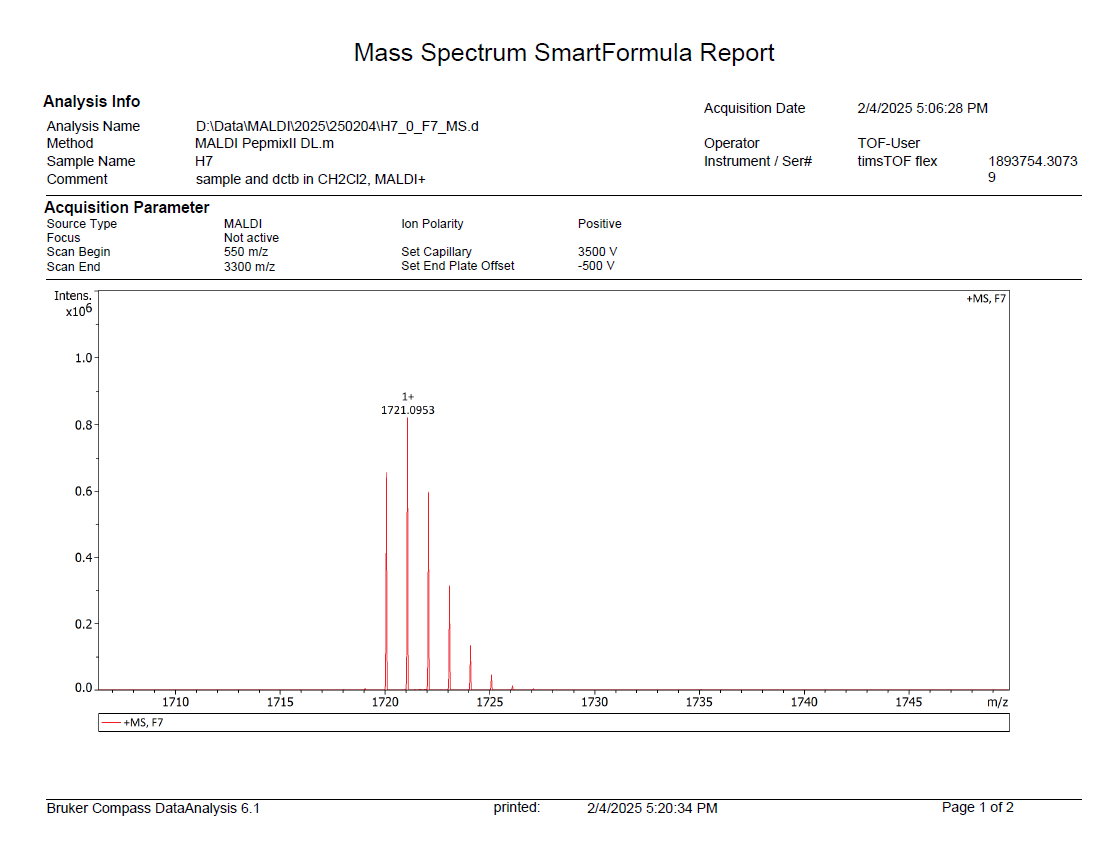


**Figure S19.** High resolution mass spectrum of **H7**.

**Figure S20.** ATR-FTIR spectrum of **H7**.
